# Low-Dose Total Body Irradiation Can Enhance Systemic Immune Related Response Induced by Hypo-Fractionated Radiation

**DOI:** 10.3389/fimmu.2019.00317

**Published:** 2019-02-27

**Authors:** Jing Liu, Jie Zhou, Min Wu, ChuanFei Hu, Juan Yang, Dong Li, Peng Wu, Yue Chen, Ping Chen, Sheng Lin, YongXia Cui, ShaoZhi Fu, JingBo Wu

**Affiliations:** ^1^Department of Oncology, The Affiliated Hospital of Southwest Medical University, Nuclear Medicine and Molecular Imaging Key Laboratory of Sichuan Province, Luzhou, China; ^2^Department of Radiation Oncology, Sichuan Cancer Hospital & Institute, Sichuan Cancer Center, School of Medicine, University of Electronic Science and Technology of China, Chengdu, China; ^3^Nuclear Medicine and Molecular Imaging Key Laboratory of Sichuan Province, Department of Nuclear Medicine, The Affiliated Hospital of Southwest Medical University, Luzhou, China

**Keywords:** systemic immune related response, hypo-fractionated radiation therapy, low-dose total body irradiation, immune enhancement, immunosuppressive microenvironment

## Abstract

A systemic immune related response (SIME) of radiotherapy has been occasionally observed on metastatic tumors, but the clinical outcomes remain poor. Novel treatment approaches are therefore needed to improve SIME ratio. We used a combination of hypo-fractionated radiation therapy (H-RT) with low-dose total body irradiation (L-TBI) in a syngeneic mouse model of breast and colon carcinoma. The combination therapy of H-RT and L-TBI potentially enhanced SIME by infiltration of CD8^+^ T cell and altering the immunosuppressive microenvironment in non-irradiated subcutaneous tumor lesions. The frequency of IFN-γ, as a tumor-specific CD8^+^ T cells producing, significantly inhibited the secondary tumor growth of breast and colon. Our findings suggest that L-TBI could serve as a potential therapeutic agent for metastatic breast and colon cancer and, together with H-RT, their therapeutic potential is enhanced significantly.

## Introduction

Radiotherapy (RT) is one of the main approaches used in cancer treatment, along with the induction of DNA damage that leads to tumor cell apoptosis. It also activates the anti-tumor immune response by exposing the tumor antigens to the host immune factors (1–3). Activation of the host immune system then leads to remissions even at sites distant from the loco-regional irradiated tissues, a phenomenon known as SIME. However, SIME induced by RT alone is rarely described, with only few published case reports. In a recent review, Reynders et al. retrieved only 23 case reports from 1973 to 2013 on the perceived SIME after RT alone (4). A common strategy of improving the SIME is to combine ionizing RT with immunotherapy (IT), which has been reported to increase the percentage of patients with abscopal tumor regression to 20% (5–7). Notably, most immunotherapeutic strategies, when used alone, failed to establish long-lasting tumor rejection in clinical trials on large patient groups (8, 9). This is most likely due to high heterogeneity of different tumor types and poor immunogenicity and evolving capability to escape immune recognition (10, 11). RT combined with IT (RT-IT) effectively changed the phenomenon (12–14). However, the repertoire is sheer endless, ranging from different RT-IT strategies including many different radiation treatments, numerous IT approaches, and choosing the right patient population and a reasonable stage of the disease. So far, no conclusive explanation could be given regarding the best strategy providing the best platform for combination approaches. Another major obstacle to precisely evaluating the effects of RT and IT combination on tumor progression is posed by the still limited available imaging modalities especially in the clinical setting (10). In addition, most patients cannot bear the costs of IT, indicating the urgent need for better strategies.

Low-dose irradiation approach, defined as ≤0.2Gy at low linear energy transfer (LET) or ≤0.05Gy at high LET, is known to induce both innate and adaptive anti-tumor immune responses (15, 16). It can activate T-cells and natural killer (NK) cells and increase T-cell proliferation, while reducing the infiltration of the immunosuppressive regulatory T-cell (Treg) in tumor tissues (17, 18). Interestingly, low-dose irradiation has been shown to inhibit or retard the development of both primary and metastatic tumors (19, 20). Since developing tumors create microenvironments that not only support neoplastic growth and metastasis but also significantly reduce the potency of both innate and adaptive anti-cancer immunity (21), the potential SIME of the combination of low-dose irradiation with RT is worth investigating.

Accumulating evidence demonstrate that the dose, mode of delivery and RT schedule are important determinants in the anti-tumor immune response, with the most vital question of “to fractionate or not to fractionate?” Due to genetic and epigenetic changes in the neoplastic cells, they may become “invisible” to immune effectors through the loss or aberrant expression of the MHC class I receptors or other molecules (22, 23). Local irradiation of tumors during standard RT can stimulate anti-cancer immunity and partially reverse the immunosuppression triggered by cancer cells. However, these effects are often induced by moderate (0.2–2.0Gy) or high (>2Gy) doses of ionizing radiation, which also harm healthy tissues, impede normal immune functions, and increase the risk of secondary neoplasms (15). Recently, Vanpouille-Box et al. revealed that single fraction doses above 12–18Gy on different cancer cells induced DNA exonuclease Trex1, which inhibits the immunogenicity of the cells by degrading their DNA that then is accumulating in the cytosol. In the Hypo-fractionated RT (H-RT), the total dose is split into large doses and administered over a short period of time (8Gy × 3), resulting in a significant increase in cytosolic dsDNA and down regulation of Trex1, which enhances the immunogenicity of colorectal and breast cancer cell lines (24, 25). Although these studies highlight the immunological effect of H-RT, as a monotherapy it rarely induces effective anti-tumor immunity that can result in systemic tumor rejection. According to the effect of low dose total body irradiation (L-TBI) in antitumor immunity, we therefore hypothesized that the combination of H-RT with our low dose total body irradiation (L-TBI) protocol might enhance the systemic anti-tumor effect and elicit the SIME as well.

Hence, in this work, we established tumors in a murine model using mouse mammary carcinoma 4T1 and colon carcinoma CT26 cells. Our results showed that tumor growth was not inhibited by L-TBI alone. Local tumor growth inhibition by H-RT did not translate into increased survival due to lung metastases and progression of the proliferation of the secondary tumor. Notably, we demonstrated for the first time that the combination of L-TBI and localized H-RT to the primary tumor activated CD8^+^ T-cell dependent anti-tumor immunity, inhibited spontaneous lung metastases and retarded secondary tumor growth, all of them significantly increasing the survival of the treated mice. These results suggested that the combination of H-RT and L-TBI might be a promising therapeutic approach for managing metastasis in cancer patients.

## Materials and Methods

### Mice

BALB/C mice (female, aged 6–8 weeks, weighing 20–25 g) were obtained from Chongqing Tengxin biotechnology Co. Ltd. (Chongqing, China). Mice were housed in standard laboratory cages under at 20–22°C, 50–60% relative humidity and 12 h light/12 h dark cycles (starting at 07:00 and 19:00, respectively), with free access to food and water. All animal experiments were approved by the Institutional Animal Care and Treatment Committee of Southwest Medical University (Luzhou, China), and all mice were treated humanely.

### Cells and Reagents

BALB/C mouse-derived mammary carcinoma 4T1 and colon carcinoma CT26 cell lines were obtained from the State Key Laboratory of Biotherapy of Sichuan University (Chengdu, China) and Army Medical University laboratory (Chongqing, China), respectively. Both cell lines were cultured in Dulbecco's modified Eagle's medium (DMEM, Hyclone, USA) supplemented with 10% fetal bovine serum (FBS; Cellmax, Australia) and 1% penicillin–streptomycin (Sigma-Aldrich, St Louis, MO, USA). Cell cultures were incubated at 37°C with 5% CO_2_ in a humidified incubator. Cells were found free of mycoplasma contamination with the help of a detection kit.

### Irradiation

All the mice were not anesthetized, positioned on a dedicated transparent radiotherapy box over the linac couch. Mice were fixed in our radiotherapy box and showed the whole right leg by a small hole, making the right leg in the tensile state and left leg natural state. All right leg and primary tumor were placed in the radiation field (Supplementary Figure 1). Our radiotherapy box has been tested by ionization chamber before radiotherapy. We tested the dose rate of the radiation field center and the middle plane. Also, we stacked in the vicinity of the tumor with thermoluminescence piece to verify dose. Radiation (L-TBI or H-RT) was delivered at a source-to-surface distance of 100 cm with a 6 MV linear accelerator (Varian Clinac 600C, USA). In this study, L-TBI was defined as a irradiation to the whole body at 0.1Gy with a dose rate of 24 cGy/min. Also local H-RT (primary tumor) was applied at 8 Gy × 3 with a dose rate of 400 cGy/min.

### Tumor Challenge and Treatment

**Figure 1:**
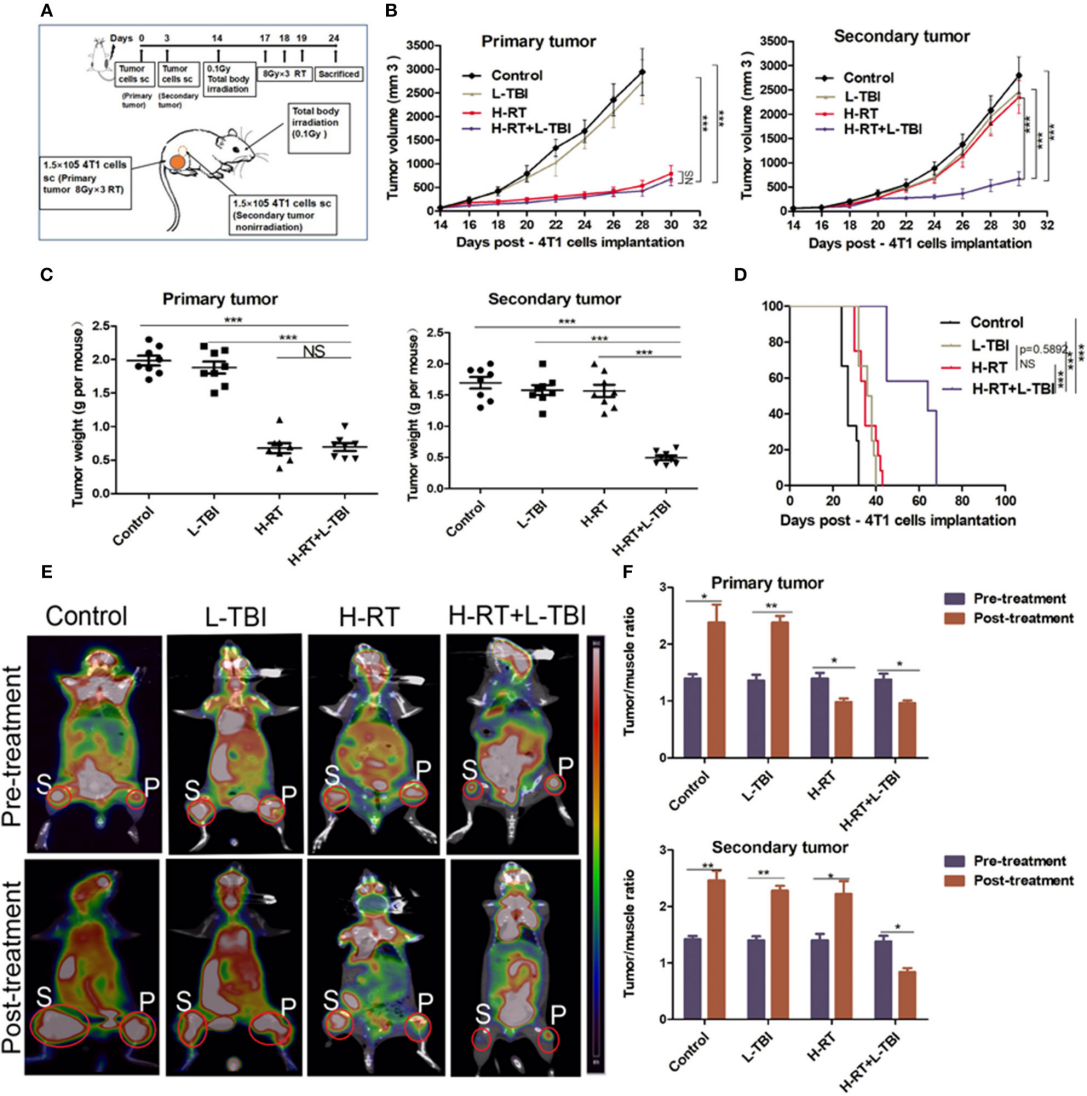
H-RT on 4T1-derived subcutaneous tumor combined with L-TBI. **(A)** Experimental groups were treated as represented in the timeline. Immunocompetent mice were injected s.c. with syngeneic 4T1 cells (1 × 10^5^) into the right (primary tumor) and left (secondary tumor) flank, respectively. H-RT was administered locally to the primary tumor from day 17 to 19, and L-TBI was administered on day 14. Primary and secondary tumor volumes were measured. On day 24, mice were sacrificed and tumors weighed. **(B)** Tumor growth of primary tumors (right panel) and secondary tumor (left panel) in mice treated with control (black line), H-RT (yellow line), L-TBI (red line), and combination of the H-RT and L-TBI (blue line). Data are the mean ± SE of 12 mice/group. **(C)** Primary tumor weight (right panel) and secondary tumor weight (left panel) on day 24 (*n* = 8 mice/group). **(D)** Overall survival of the tumor bearing mice of different treatment groups (*n* = 12 mice/group). **(E)** Representative pre- and post-treatment 18F-FDG PET images of tumor-bearing mice in control, and treatments groups (L-TBI, H-RT, H-RT+L-TBI; *n* = 5 mice/group). **(F)** Tumor/muscle ratio of primary (right panel) and secondary (left panel) tumor in the pre-treatment (on day 13) and post-treatment (on day 24) period (*n* = 5 mice/group). The experiment has been repeated in similar result (^*^P < 0.05, ^**^P < 0.01, ^***^ *P* < 0.001 and NS = not significant).

4T1 mammary carcinoma cells (1.5 × 10^5^) and CT26 colon carcinoma cells (2.5 × 10^4^) were subcutaneously injected in the right flank of each BALB/C mouse on day 0 separately. Also the same amount of cells were injected in the contralateral flank on day 3. The tumor arising from day 0 inoculum was designated as “primary” tumor and was irradiated, while the “secondary” tumor from the second inoculum was not irradiated (Figure 1A). On day 14, when the primary tumor reached an average size of 60–80 mm^3^, mice were randomly divided into four groups according to the RT administered: (a) control group: non-irradiated; (b) L-TBI: low-dose total body irradiation at 0.1 Gy on day 14; (c) H-RT: 3 doses of localized radiations at 8 Gy each dose on the primary tumor on day 17, 18, and 19; (d) H-RT+L-TBI: L-TBI on day 14 followed by H-RT on day 17–19. Tumor size was monitored every 2 days, and tumor growth or regression was recorded. The perpendicular diameter of each tumor was measured using Vernier calipers, and tumor volume was calculated using the following formula: length × width^2^ × 0.52, by two researcher independently (26, 27). On day 24, some of mice were anesthetized and sacrificed by cervical dislocation. The requisite organs were harvested and processed for further analysis. The remaining mice were used to observe survival and make survival curves. Meanwhile, we measured the tumor volume of these mice until death. When the tumor volume exceeded 4 cm^3^, all mice were sacrificed.

### Measurement of Lung Surface Nodules

After sacrificing the mice on day 24 post-inoculation, their lungs were resected and fixed in 10% neutral-buffered formalin for 24 h. The pulmonary metastatic nodules were counted and their diameters were measured under a dissecting microscope. The nodules were classified into 4 levels according to their diameter as follows: I. < 0.5 mm, II. 0.5–1 mm, III. 1–2 mm, and IV. >2 mm. Then, the lung surface transfer nodule was calculated using the formula: I × 1 + II × 2 + III × 3 + IV × 4 (28). As regard histopathological examination, the fixed tissues were embedded in paraffin, sectioned, and stained with hematoxylin and eosin (H&E), according to standard protocols. Microscopically analysis of all the slides was performed by a light microscopy (Olympus Cor, Tokyo, Japan) linked to computerized image system (Image-Pro Plus V6.0, Silver Spring, MD).

### Micro 18F-FDG PET/CT Imaging

The early effects of different treatments were evaluated using micro PET/CT scans and all images were analyzed by using an Inveon micro PET/CT animal scanner (Siemens, Germany). Mice were fasted for 12 h and then anesthetized by intraperitoneal injection with 1% pentobarbital (5 ml/kg). Mice were then placed in the center of the scanner, intravenously injected with 200–300 μCi FDG, and then scanned. PET/CT images were exported one h after injection of 18F-FDG trace. The parameters used for PET/CT scanning were as follows: 80 kV, 500 μA, slice thickness of 1.5 mm, and 10 min per bed position.

The image plane with the largest tumor appearance on the PET/CT fusion image was selected for analysis, and the irregular region of interest (ROI) covering the entire tumor was manually drawn. ROIs were also drawn on the paraspinal muscles. The tracer uptake value in both the tumor and muscle tissue was determined in the attenuation-corrected transaxial tomographic slices by calculating the standard uptake value (SUV), and was measured by means of ROI. The 18F-FDG maximum SUV of each lesion was obtained from the selected ROI and then compared to the SUVs of the contralateral paraspinal muscles to calculate the tumor/muscle (T/M) ratio.

### Flow Cytometry Analysis

The breast cancer tumors were resected, and then homogenized in 0.2% collagenase type IV, 0.01% hyaluronidase, and 0.002% DNase I (all enzymes from Solarbio science, Beijing, China) in DMEM medium at 37°C for 40 min. Also, spleen tissue was resected, grinded and filtered into a single cell suspension, according to standard protocols. The blood cell lysate kits were used for removing red blood cells (BD Biosciences, CA, USA). The single cell suspension thus obtained was stained with the fixable viability stain 780, and then the harvested cells were labeled with the following antibodies: CD45-PerCP, CD11b-APC, Gr1-FITC, Siglec-F-PE, Ly6G-PE-Cy7, Ly6c-FITC, CD11c-PE, F4/80-APC/Cy7, CD206-FITC, CD3-PerCP-Cy5.5, CD4-FITC, CD8-PE-Cy7, CD86-FITC, and INF-γ-APC antibodies according to the manufacturer's protocol (BD Bioscience, CA, USA). For INF-γ staining, cells were stimulated *in vitro* with a cell stimulation cocktail (plus protein transport inhibitors) (BD Bioscience) for 6 h. After surface labeled with CD3-PerCP-Cy5.5 and CD8-PE-Cy7 antibodies, cells were then processed using a fixation and permeabilization kit (BD Bioscience) and stained with antibodies from BD to IFN-γ. In order to identify the frequencies of CD8^+^ cell, mouse anti-CD8/Lyt2.1 monoclonal antibody (clone HB129/116-13.1) and corresponding isotype control (clone C1.18.4) were purchased from BioXcell (West Lebanon, NH, USA). The 4T1-bearing mice were intraperitoneally treated with 400 μg of anti-CD8/Lyt2.1 monoclonal antibody and isotype control as described in Supplementary Figure 4A. The stained samples were analyzed using a Beckman Coulter Gallios flow cytometry (Beckman Coulter, Miami, FL, USA). All flow cytometry data were analyzed with FlowJo software (version 10.0). Isotype-matched control antibodies were all purchased from BD (BD bioscience, CA, USA) and used at the same concentration as test antibodies. Fluorescence minus one (FMO) controls was used for determining the percentage of positive cells.

### Immunohistochemistry

Tumor tissues were fixed in 10% neutral-buffered formalin and embedded in paraffin, and 4 μm thick sections were cut and used for immunohistochemistry (IHC). The sections were labeled with the following antibodies: gamma-H2AX, TUNEL, CD3, and CD86, according to the manufacturer's instructions (Bioworld Technology, Nanjing, China). Images were taken using an optical microscope (Olympus, Tokyo, Japan). For each tumor section, the total number of cells and those positive for gamma-H2AX, CD3, and CD86 were counted in five randomly selected fields (original magnification × 200), and the percentage of positively stained cells was calculated. Similarly, TUNEL-positive brown nuclei were also counted, and the percentage of apoptotic cells per field was calculated.

### ELISA Measurements

Levels of INF-γ were measured by standard ELISA method by specific-antibody ELISA kits according to the manufacturer's instructions (Cheng Lin biotechnology, Beijing, China). In details, 0.5 mL of the blood samples were collected from the retro-orbitally sinus on day 24 post inoculation. Blood samples were left undisturbed at room temperature (20–25°C) for 20 min, and then were centrifuged at 2,000 g for 20 min. The serum was aspirated under sterile conditions and was stored at −80°C till further analysis.

### Statistical Analysis

All statistical analysis were performed using SPSS 17.0 software (Chicago, Illinois, USA). Comparisons between two groups were made using Student's *t*-test, as well as one-way or two-way analysis of variance (ANOVA) was used for more than two groups. Survival curves were plotted based on the Kaplan-Meier method. Data are presented as mean ± standard error (SE). For all tests, two-sided *p* < 0.05 and high statistical significance at < 0.01 and < 0.001 were considered statistically significant. All charts were designed by Prism 5.0 (GraphPad, La Jolla, CA, USA).

## Results

### L-TBI (0.1 Gy) Combined With H-RT (8 Gy × 3) Suppressed the Primary Tumor, and Effectively Inhibited the Secondary Tumor

BALB/C-derived mammary carcinoma 4T1 cells were used to establish a tumor model in order to test whether local H-RT can trigger systemic antitumor effects outside the radiation field when combined with L-TBI. According to a reported research that the dose of 0.1 Gy total body irradiation can enhance immune effect (29), mice were subjected to total body irradiation at 0.1Gy. We induced subcutaneous tumors in the mice at two separate sites: the primary tumor was irradiated by H-RT to determine the direct therapeutic effect of H-RT±L-TBI, while the secondary tumor was not irradiated and served to measure the potential indirect, systemic effect of H-RT± L-TBI (Figure 1A).

L-TBI alone did not delay the growth of either the primary or secondary tumors, as the tumor volume did not significantly change compared with the non-irradiated control group (*P* > 0.05). In line with the previous reports, H-RT indeed led to a significant growth delay of the irradiated primary tumors (*P* < 0.001 from day 18) but did not have a SIME on secondary tumors. Of note, we found that the combination of L-TBI and H-RT significantly delayed the growth of both the primary and secondary tumors (*P* < 0.001 from day 22; Figure 1B). Consistently, the weight of the harvested abscopal tumors was also significantly reduced in the combination therapy group compared to the others (with complete regression in 2 mice; *P* < 0.001), while reduction in primary tumor weight was similar in the H-RT and H-RT+L-TBI groups (Figure 1C). Taken together, local H-RT combined with L-TBI showed the highest tumor inhibitory effect and SIME was also elicited. The anti-tumor efficacy of H-RT+L-TBI translated to the best overall survival. H-RT+L-TBI treated mice showed a median survival time of 64 days compared to the 35 days in H-RT, 37 days in L-TBI, and 27 days in the control group (*P* < 0.001; Figure 1D).

Micro 18F-FDG PET/CT imaging (representative images in Figure 1E) showed significant differences between the pre-treatment and post-treatment T/M values within all four groups (Figure 1F). The primary tumor of the H-RT+L-TBI and H-RT group showed a significant decrease in the T/M values following treatment. In contrast, the post-treatment T/M values of secondary tumors showed a significant decrease only in the H-RT+L-TBI group, further indicating a better systemic anti-tumor response (SIME) of H-RT and L-TBI combination.

### Impact of the Duration and Sequence of Combination Therapy on SIME

**Figure 2:**
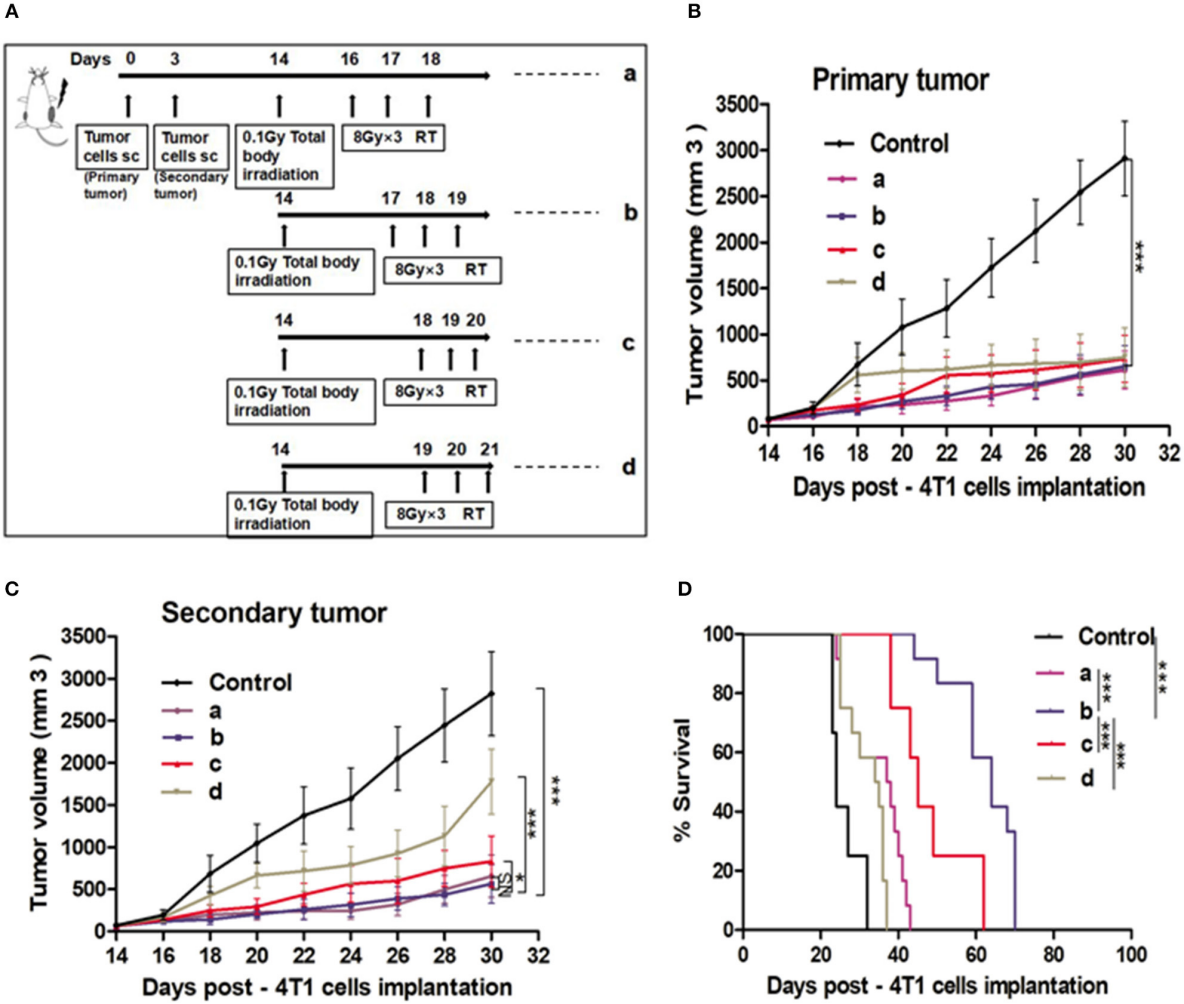
Effect of different time intervals of the H-RT and L-TBI combination therapy in 4T1 tumor-bearing mice. **(A)** Treatment timeline of the 4T1 mammary carcinoma BALB/C mouse model. Tumor growth of primary tumors **(B)** and secondary tumors **(C)** in different experimental groups. **(D)** Overall survival curves of the treatment groups. Immunocompetent mice were injected s.c. with syngeneic 4T1 cells (1 × 10^5^) into the right (primary tumor) and left (secondary tumor), respectively. The 12 mice/group irradiated with H-RT (8 Gy x 3) at 48 h (a), 72 h (b), 96 h (c), and 120 h (d) after L-TBI. Primary and secondary tumor volumes were measured. Data are expressed as mean ± SE (^*^P < 0.05, ^***^P < 0.001, and NS = not significant).

To determine whether the post L-TBI interval could impact the therapeutic effect of the combination therapy, we started the local H-RT at 48, 72, 96, and 120 h after L-TBI (scheme shown in Figure 2A). Compared to the non-irradiated control, the primary tumor volume of the other groups showed a significant decrease regardless of the post L-TBI interval, while the maximum growth delay of the secondary tumor was achieved by the administration of H-RT at 48 and 72 h after L-TBI before 30 days (Figures 2B,C). In addition, local H-RT 72 h after L-TBI therapy led to the best overall survival (Figure 2D).

To test the therapeutic impact of the sequence of the combination therapy, we administered local H-RT 3 days before L-TBI (b-L-TBI), 3 days after L-TBI (a-L-TBI), or simultaneously with L-TBI (s-L-TBI) (Supplementary Figure 2A). a-L-TBI achieved the best therapeutic effect represented by a significant tumor growth delay and improved survival of the treated mice (Supplementary Figures 2B–D).

### Effect of Combination Therapy on Apoptosis

**Figure 3:**
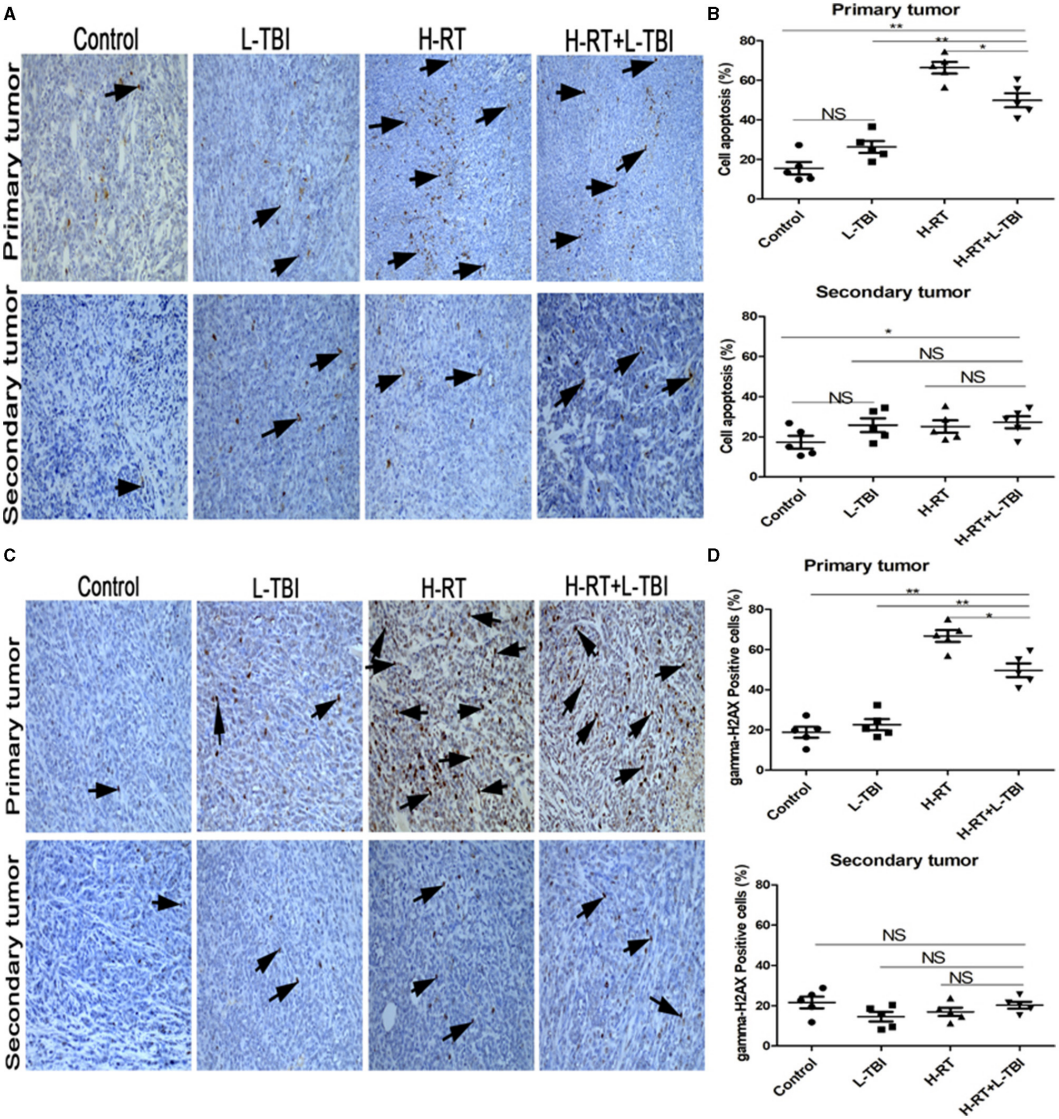
Effect of combination H-RT and L-TBI therapy on apoptosis in 4T1 tumor-bearing tissues. **(A)** Comparison of representative TUNEL IHC-stained in different treatment groups. **(B)** Percentage of TUNEL positive cells in the primary and secondary tumor. **(C)** Representative gamma-H2AX IHC staining image in different treatment groups. **(D)** Percentage of gamma-H2AX positive cells in the primary and secondary tumor. The arrows point to the TUNEL and gamma-H2AX positive cells in the tumor tissue (original magnification × 200). Data are expressed as mean ± SE of 5 mice/group. (^*^P < 0.05, ^**^ *P* < 0.01, and NS = not significant).

RT is known to induce apoptosis of cancer cells. To determine whether the direct and abscopal anti-tumor effect of the combined therapy was also related to apoptosis, tumor tissue sections were stained with TUNEL. Compared to the sporadic apoptotic cells seen in the primary tumor in the non-irradiated control and the L-TBI treated group, a significantly higher number of apoptotic cells was observed in the H-RT and H-RT+L-TBI group (Figure 3A). However, the primary tumor of the H-RT group showed a higher apoptosis rate than the tumor of the H-RT+L-TBI group (*P* < 0.05; Figure 3B). In contrast, little apoptosis was observed in the secondary tumor in all groups (*P* > 0.05; Figure 3B). Taken together, the percentage of apoptotic cells in the primary tumor was dramatically higher in the H-RT group compared to the others, while apoptosis was not the main underlying mechanism of the anti-tumor immune response.

To determine whether DNA damage mediated the primary and secondary tumor growth inhibition, gamma-H2AX staining was performed on the tumor tissue (Figure 3C). A significantly higher number of gamma-H2AX positive cells were seen in the primary tumor tissue of the H-RT and H-RT+L-TBI group compared to the L-TBI and control group, while H-RT induced significantly more gamma-H2AX foci compared to H-RT+L-TBI (*P* < 0.05; Figure 3D). However, in the secondary tumor, very low level of gamma-H2AX staining was observed in all groups (*P* > 0.05; Figure 3D). In conclusion, H-RT resulted in more DNA damage compared to H-RT+L-TBI. Therefore, L-TBI reduced DNA damage caused by H-RT.

### Increased Secondary Tumor Infiltration of CD8^+^ T-Cells After H-RT+L-TBI Is Probably Dependent on IFN-γ

**Figure 4:**
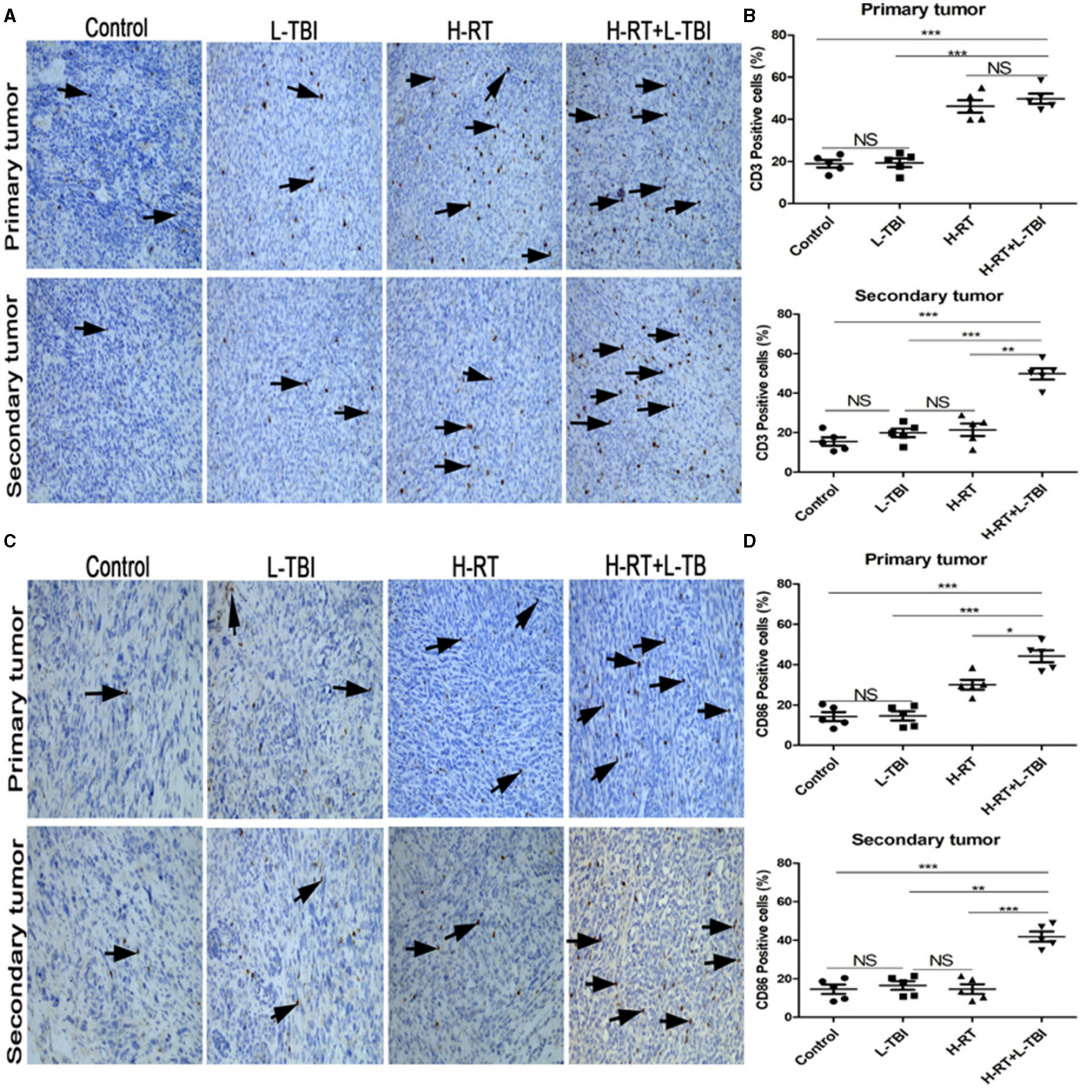
Comparison of CD3^+^ and CD86^+^ lymphocytes in different treatment groups. **(A)** Representative images of CD3 IHC in tumor tissues of different treatment groups. **(B)** Percentage of CD3 positive cells in the primary and secondary tumor. **(C)** Representative IHC images of CD86 infiltration in the tumor tissue of different treatment groups. **(D)** Percentage of CD86 positive cells in the primary and secondary tumor. The arrows point the CD3 and Cd86 positive cells in tumor tissues from mice that received different treatments (original magnification × 200). Data are expressed as mean ± SE of 5 mice/group. (^*^P < 0.05, ^**^P < 0.01, ^***^P < 0.001, and NS = not significant).

Since irradiation triggers an immune response, we also assessed the infiltration of CD3^+^ and CD86^+^ lymphocytes in the primary and secondary tumor tissue (Figures 4A,C). The primary tumor of the H-RT and H-RT+L-TBI group showed a higher percentage of CD3^+^ cells compared to the L-TBI and control group, and no significant difference was observed between H-RT+L-TBI and H-RT group (Figure 4B). Furthermore, the percentage of CD86^+^ cells in the primary tumor was the highest in the H-RT+L-TBI group (Figure 4D). A significantly increased number of CD3^+^ and CD86^+^ positive cells were seen in the secondary tumor of the H-RT+L-TBI group compared to the other groups (Figures 4B,D). Due to activated tumor-associated CD11c^+^DCs, which higher expression of CD86, we further evaluated the expression of tumor-associated CD86^+^DCs (CD45^+^CD11b^+^CD11c^+^CD86^+^) within the tumor tissue by flow cytometry. In the secondary tumor, the number of CD86^+^DC cells was significantly increased after combination therapy (Supplementary Figures 3A,B).

**Figure 5:**
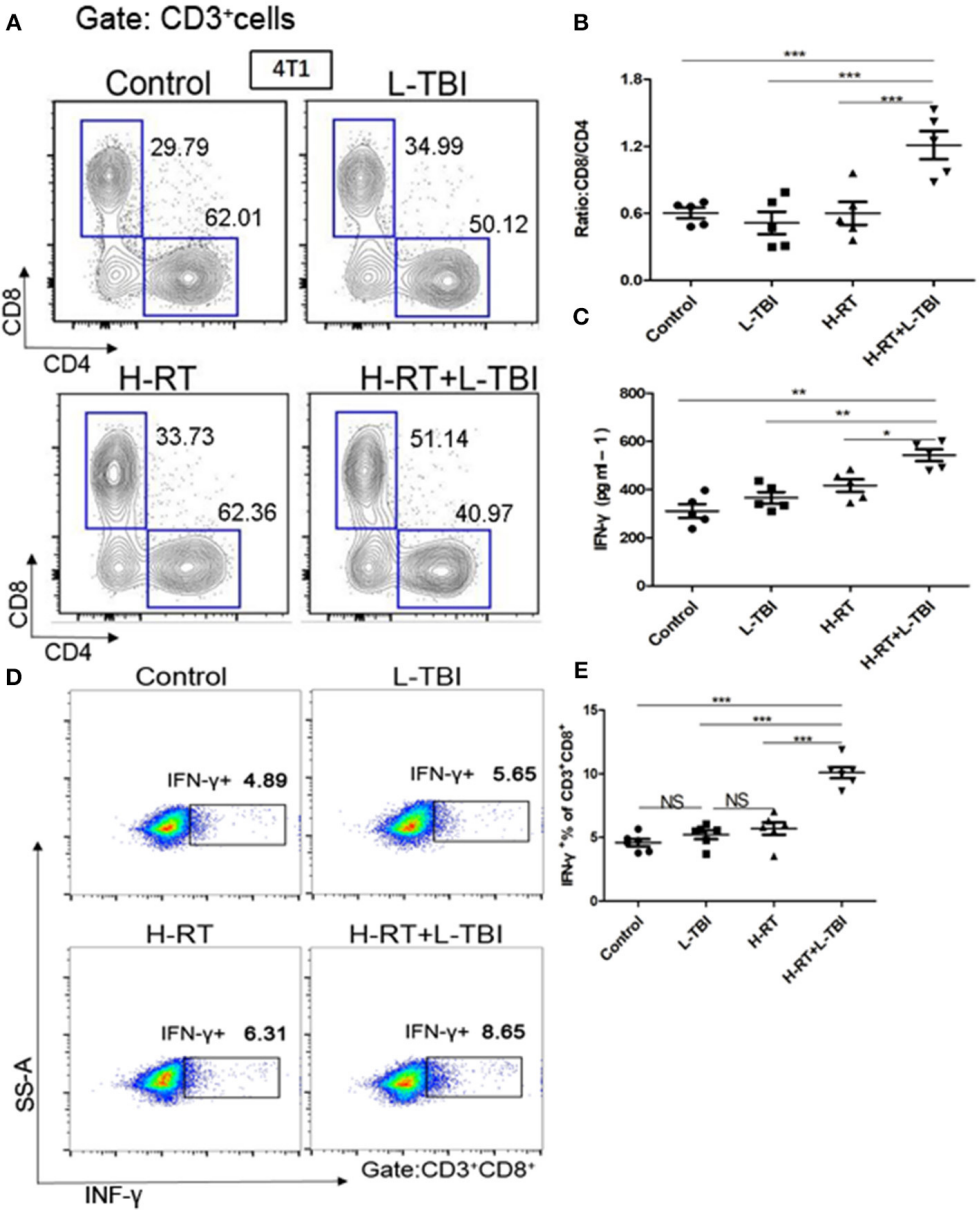
Expression of CD8^+^/CD4^+^ T cells in mice treated with H-RT and L-TBI radiotherapy. **(A)** The frequencies of CD8^+^ and CD4^+^ cells induced in the secondary tumor of each group (*n* = 5 mice/group). **(B)** Ratio of CD8^+^/CD4^+^ cells in the secondary tumor of each group (*n* = 5 mice/group). **(C)** ELISA results of the INF-γ levels (pg/ml) in various groups (*n* = 5 mice/group). **(D)** Representative dot plots of CD3^+^CD8^+^INF-γ^+^ cells in the secondary tumor tissue of control, L-TBI, H-RT, and H-RT+L-TBI group (*n* = 6 mice/group). **(E)** Comparison plot of CD3^+^CD8^+^INF-γ^+^ cells in the secondary tumor tissue of different various groups (*n* = 6 mice/group). The cells were gated on living lymphocytes and then on CD8^+^ and CD4^+^ cells and the percentages of CD3^+^CD8^+^INF-γ^+^ T-cells were determined by flow cytometry analysis. Data are representative charts or the percentages of individual subjects. The lines indicate median values for each group. (^*^P < 0.05, ^**^P < 0.01, ^***^P < 0.001, NS = not significant).

Combination therapy increased activated CD8^+^ T cells in the secondary tumor. A dramatic increase of infiltrating CD8^+^ T-cells in the secondary tumor of the H-RT+L-TBI group (Figures 5A,B), suggesting that cell-mediated immunity was responsible for the SIME of the combined RT. Since tumor-infiltrating CD8^+^ T-cells induce anti-tumor immune response via cytokines such as IFN-γ (30–32), we assessed the levels of IFN-γ in the mouse serum by ELISA. H-RT+L-TBI led to a significant increase in IFN-γ levels (Figure 5C). In order to identify the frequencies of CD8^+^ IFN-γ, we performed intracellular CD3^+^CD8^+^IFN-γ^+^ staining (Figure 5D). The combination therapy increased the number of IFN-γ^+^CD8^+^T cells in the secondary tumor (Figure 5E), confirming the induction of tumor-specific immune response. Collectively, these results demonstrate that the combined treatment with H-RT and L-TBI induced tumor-specific T cell responses that, when sufficiently strong, could result in complete remission of abscopal tumors.

CD8^+^T cells were indispensable for SIME with combination therapy. To confirm that tumor-specific CD8^+^T cells induced by combination therapy contributed to growth suppression of distant metastatic tumors, CD8^+^ cells were depleted by anti-CD8/Lyt2.1 monoclonal antibody (Supplementary Figure 4A). The tumor volume was statistically not significant in either the primary or the secondary tumors between control and H-RT+L-TBI after the percentage of CD8^+^ T cells decreased (Supplementary Figures 4B,C). We confirmed depletion of CD8^+^ cells using flow cytometry (Supplementary Figure 4D). The result showed that the decrease of CD8^+^ cells ended the suppressive effects of the combination therapy in both primary and secondary tumors.

### H-RT+L-TBI Altered the Immunosuppressive Microenvironment of Secondary Tumors

**Figure 6:**
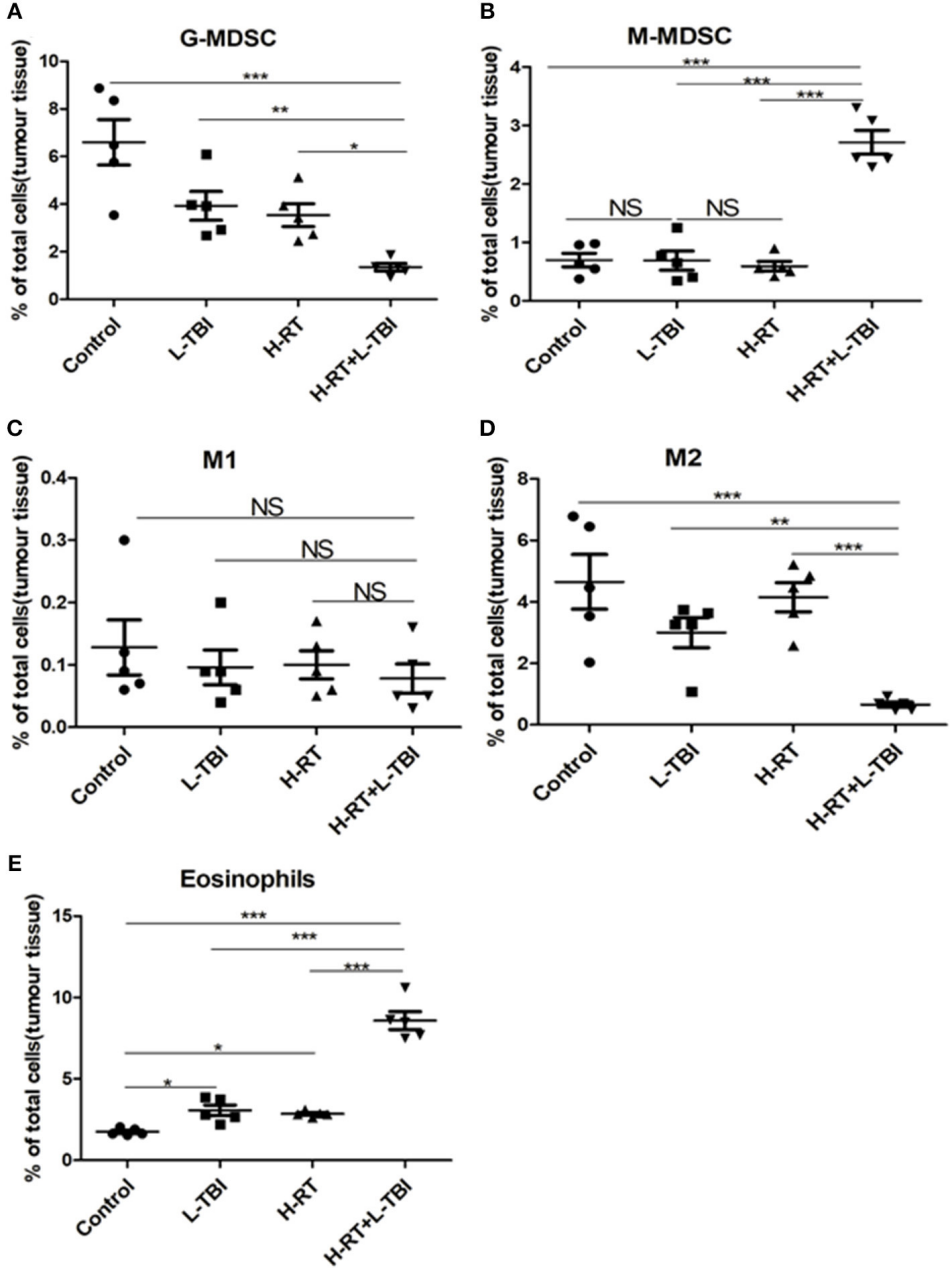
Immunosuppressive microenvironment effects of H-RT and L-TBI combination therapy on 4T1 tumor-bearing mice. The percentage of the G-MDSCs (defined as CD45^+^CD11c^−^CD11b^+^Ly6G^+^Ly6c^low^) **(A)**, M-MDSCs (defined as CD45^+^CD11c^−^CD11b^+^Ly6G^−^Ly6c^hi^) **(B)**, M1 (defined as CD45^+^CD11b^+^F4/80^+^ CD206^−^) **(C)**, M2 (defined as CD45^+^CD11b^+^ F4/80^+^CD206^+^) **(D)**, and Eosinophils (defined as Siglec-F^+^Gr1^low^) **(E)** were analyzed by flow cytometry analysis. Data are representative charts or the percentages of individual subjects. Data are expressed as mean ± SE of 5 mice/group. The statistical significance of differences was determined by ANOVA. (^*^P < 0.05, ^**^P < 0.01, ^***^P < 0.001, and NS = not significant).

To further explore the underlying mechanism of the anti-tumor effect of the combined RT, we investigated the secondary tumor microenvironment in the different groups. Large solid tumors can evade anti-tumor immunity partly by inducing an immunosuppressive/tolerogenic microenvironment that includes regulatory cells such as myeloid-derived suppressor cells (MDSCs), tumor-associated macrophages (TAMs), and regulatory CD4^+^ T-cells (Tregs) (33–38). Therefore, we analyzed these populations in the tumor tissues by flow cytometry (Supplementary Figures 5, 6). The percentage of the granulocyte (G)-MDSCs was the lowest and that of the monocytic (M)-MDSCs was the highest within the total cell population in the H-RT+L-TBI group, (*P* < 0.001; Figures 6A,B). In addition, the tumor of the L-TBI, H-RT and control group showed an increase in the number of G-MDSCs post treatment, while the proportion of M1 cells in the total cell population was similar in all groups (Figure 6C), and the proportion of M2 cells was the lowest in the H-RT+L-TBI group (Figure 6D). In contrast, treatment with L-TBI or H-RT alone led to an increase in the percentage of M2 cells. Taken together, the combination treatment reversed the immunosuppressive tumor microenvironment (TME) in the distant tumor by reducing the percentage of G-MDSCs and M2 cells. Since eosinophil infiltration is associated with tumor inhibition, we also examined the percentage of Eosinophils (Siglec-F^+^Gr1^lo^) within the tumor tissue (Supplementary Figure 7). Both L-TBI and H-RT treatment led to an increase of eosinophil population. Notably, such an expansion was further increased by L-TBI+H-RT combination therapy (Figure 6E). Taken together, the combination treatment reversed the immunosuppressive tumor microenvironment (TME) in the distant tumor by reducing the immunosuppressive G-MDSCs and M2 macrophages and increased the percentage of anti-tumor eosinophil population.

### H-RT+L-TBI Inhibited 4T1 Lung Metastasis

**Figure 7:**
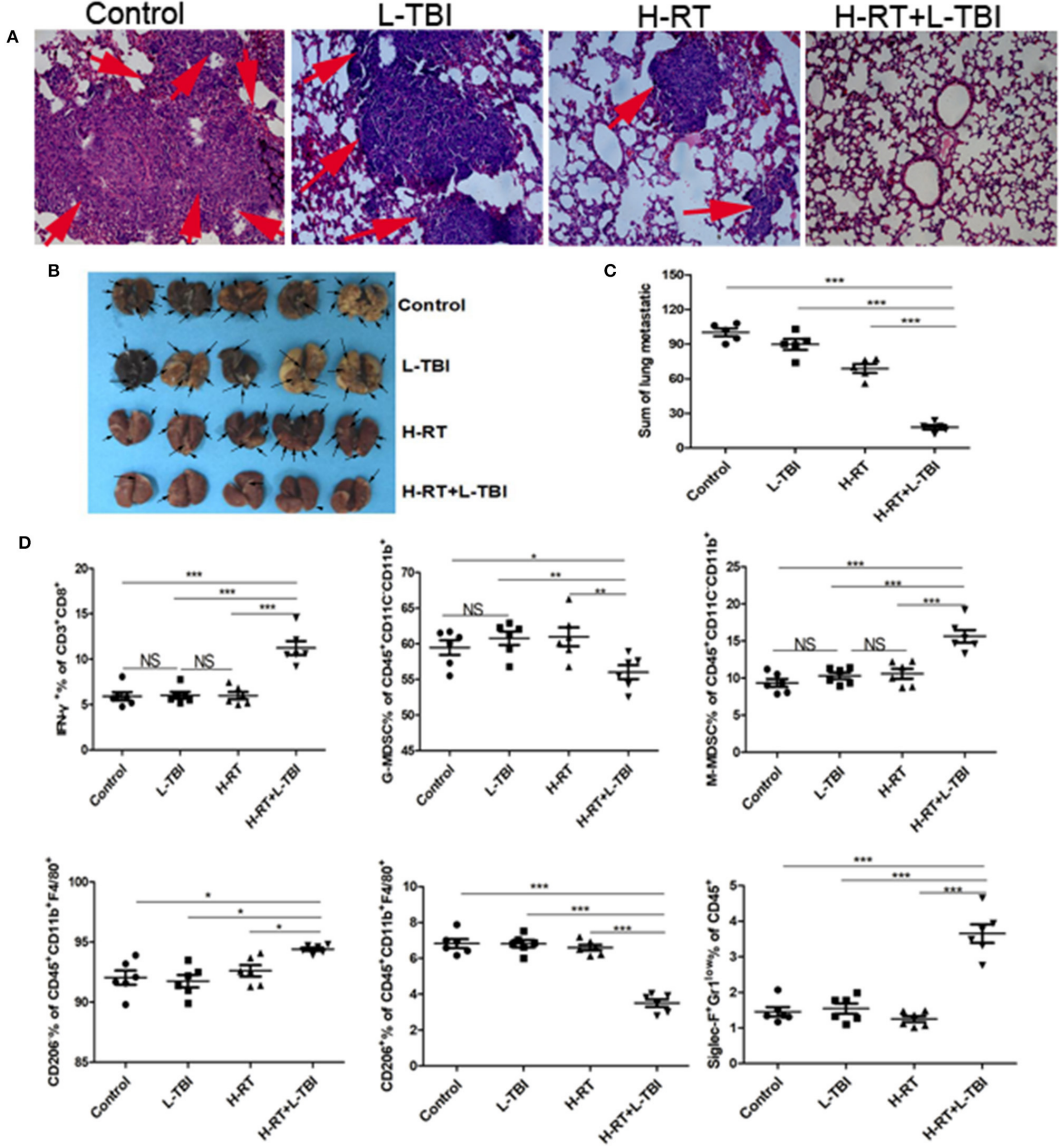
Anti-metastatic effect of the H-RT and L-TBI combination therapy. **(A)** Comparison of representative H&E-stained in lung tissue sections at 24 days after 4T1 cells implantation (original magnification × 100). The arrows point to the metastatic infiltration. **(B)** Representative macroscopic images of the lungs in different groups. The arrows point to the metastatic nodules in the lung (*n* = 5 mice/group). **(C)** Comparison of the lung metastatic nodules between control, L-TBI, H-RT, and H-RT+L-TBI group (*n* = 5 mice/group). **(D)** Frequency of IFN-γ^+^ CD8^+^ T cells (CD3^+^CD8^+^IFN-γ^+^), G-MDSC (CD45^+^CD11c^−^CD11b^+^Ly6G^+^Ly6c^low^), M-MDSC (CD45^+^CD11c^−^CD11b^+^Ly6G^−^Ly6c^hi^), M1 (CD45^+^CD11b^+^F4/80^+^CD206^−^), M2 (CD45^+^CD11b^+^F4/80^+^CD206^+^), and Eosinophils (CD45^+^Siglec-F^+^Gr1^low^) in mice spleens (*n* = 6 mice/group). Data are representative charts or the percentages of individual subjects. Data are expressed as mean ± SE. In general, combined therapy of H-RT+L-TBI significantly reduced the number and diameter of lung metastatic nodules (*P* < 0.001). (^*^P < 0.05, ^**^P < 0.01, ^***^P < 0.001, NS = not significant).

The murine 4T1 tumor closely resembles human breast cancer both in terms of immunogenicity and metastasis. Since 4T1 cells primarily metastasize to the lungs, we examined the lungs for metastatic nodules and tumor cell infiltration. In addition to considerably less metastatic infiltration (Figures 7A,B), H-RT+L-TBI mice had significantly fewer and smaller lung metastatic nodules (*P* < 0.001; Figure 7C). Three of the 5 H-RT+L-TBI mice had no visible nodules larger than 2 mm. Thus, the combination therapy significantly inhibited lung metastases, which was most likely the reason for improved survival.

Combination therapy induces SIME. To further confirm the effect of the combination therapy on the systemic immune system, we observed the number of IFN-γ^+^CD8^+^T cells, G-MDSC, M-MDSC, M1, M2 and Eosinophils in the spleen from different groups (Supplementary Figure 8). The combination treatment reduced the percentage of G-MDSCs and M2 cells and increased the percentage of anti-tumor eosinophil and IFN-γ^+^CD8^+^ T cell population in the spleen (Figure 7D). Taken together, the combination treatment induced systemic immune related responses.

### 4T1 Breast Tumor Responded to Accelerated L-TBI in a Manner Similar to CT26 Tumor

**Figure 8:**
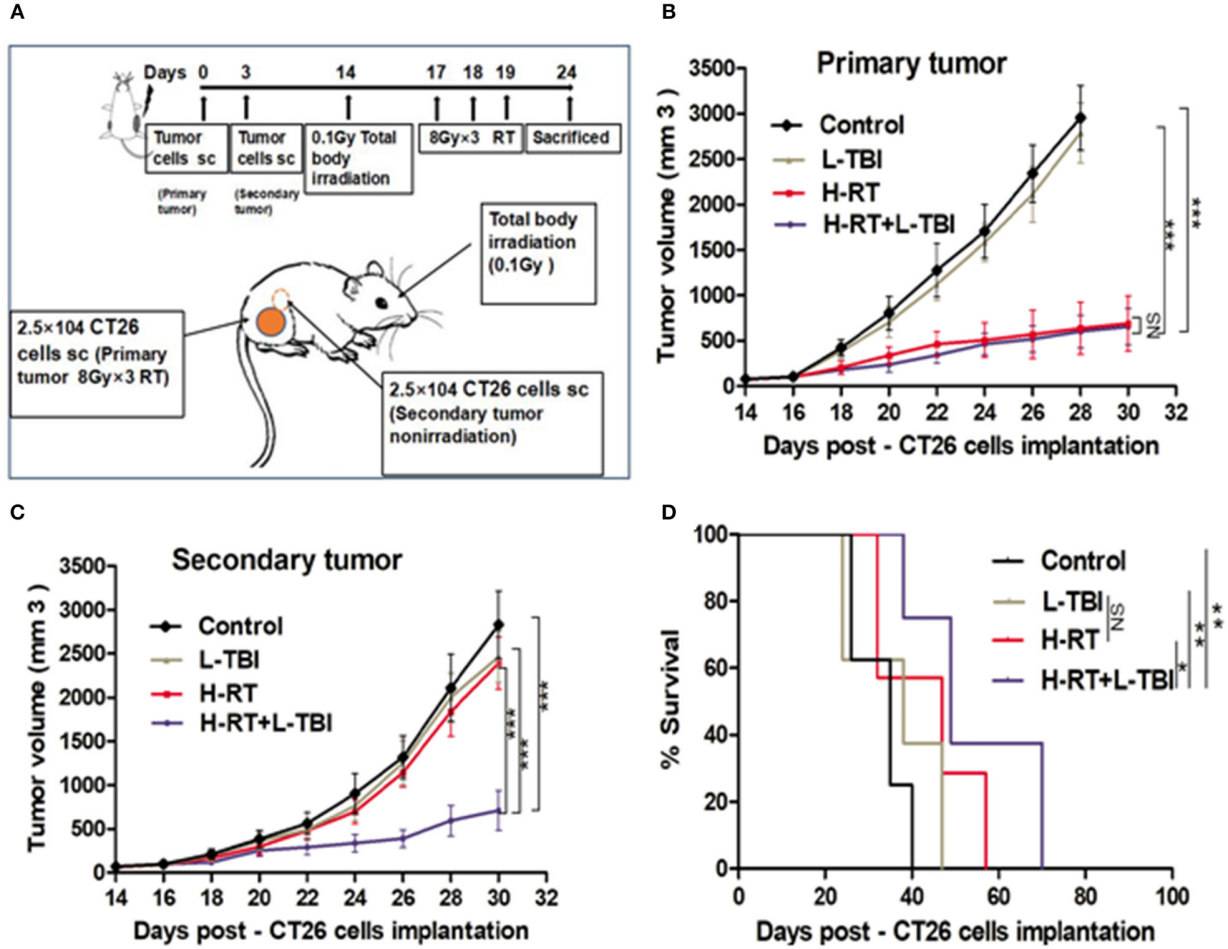
Combination therapy of CT26 tumor with H-RT and L-TBI. **(A)** CT26-derived tumors model and treatment timeline. Immunocompetent mice were injected s.c. with syngeneic CT26 cells (2.5 × 10^4^) into the right (primary tumor) and left (secondary tumor) flank, respectively. Only the primary tumor received H-RT (*n* = 12 mice/group). CT26 tumor growth curves of primary irradiated tumors **(B)** and secondary non-irradiated tumors **(C)** between different groups (*n* = 8 mice/group). **(D)** Overall survival curves of investigation groups (*n* = 8 mice/group). (^*^P < 0.05, ^**^P < 0.01, ^***^P < 0.001, and NS = not significant).

To determine whether the efficacy of H-RT+L-TBI was dependent on the tumor type and/or genetic background of the mice, we established another tumor model in BALB/C mice using the murine CT26 colon carcinoma cells, and subjected them to the same RT protocols (Figure 8A). As observed in the 4T1 model, L-TBI did not have any effect on the growth of primary or secondary CT26 tumor, H-RT caused a significant growth delay only in the primary tumor (*P* < 0.001), while the combined treatment significantly inhibited the growth of both primary and secondary tumor (Figures 8B,C). Therefore, H-RT+L-TBI triggered a SIME in the CT26 model as well. In addition, a 80-day follow-up showed a significant survival benefit in mice treated with H-RT+L-TBI as compared to H-RT alone (*P* < 0.001; Figure 8D). However, we could not observe a survival benefit using L-TBI alone.

## Discussion

To the best of our knowledge, this is the first report to demonstrate that H-RT (8 Gy × 3) combined with L-TBI (0.1 Gy) enhanced the systemic or abscopal anti-tumor effect of RT, in addition to the other local effects of irradiation. The enhanced therapeutic efficacy was manifested by increased primary tumor regression and decreased metastasis, resulting in improved survival. These findings indicate that this novel combination approach could potentially control metastasis in advanced cancer patients.

We also observed an L-TBI-induced adaptive immune response by sequential H-RT treatment in this mouse model. L-TBI administration before H-RT not only protected the immune system of the mice, but also resulted in a maximum inhibition of primary tumor growth compared to the other groups. Interestingly, when the immune function was impaired by b-L-TBI, the primary tumor could still be inhibited to some extent. The therapeutic effect of simultaneous administration of L-TBI and H-RT was similar to that of b-L-TBI. TUNEL and gamma-H2AX staining showed that H-RT alone and L-TBI+H-RT could both inhibit primary tumor growth by inducing apoptosis and DNA damage, while the combination treatment induced less apoptosis and DNA damage than H-RT alone. Therefore, we speculated that another reason might induce this phenomenon, such as the immune effect. The combination treatment resulted in CD8^+^ T-cells, IFN-γ^+^CD8^+^ T cells and DCs infiltration in the non-irradiated tumors as well, resulting in a marked attenuation of tumor growth. However, in L-TBI alone and H-RT alone group, these two treatments did not delay the growth of the non-irradiated tumor. It was the improved immune response that played a key role in L-TBI+H-RT induced abscopal tumor inhibition. This indicated that L-TBI was the key determinant of SIME via induction of the adaptive immune response. These findings emphasize the importance of the immune response in tumor RT, and might help to promote the application of low dose RT as a novel approach in treating metastasis.

RT alone rarely induces SIME because the tumor microenvironment not only support neoplastic growth and metastasis, but also inhibits host anti-cancer immunity through various strategies (39, 40). Growth of the 4T1 and CT26 tumor is accompanied with increased MDSCs, TAMs and Treg cell population, which have immunosuppressive functions (41–43). MDSCs, especially the G-MDSCs, enable tumor immune escape by inhibiting the activation of T-cells, DCs and NK cells (44). MDSCs also promote tumor metastasis and progression (45, 46). The tumor associated macrophages (TAMs) are classified into the classic/pro-inflammatory M1 and the anti-inflammatory M2 macrophages. M1 are cytotoxic cells that identify tumor antigen through antigen presentation, and kill the tumor cells. M2 inhibit T-cell and NK cell activation and proliferation, and inhibit the anti-tumor immune response by producing anti-inflammatory factors such as IL-10, TGF-β and prostaglandin E2 (43, 44, 47). The balance between immunosuppression and activation ultimately results in a successful tumor elimination. Due to the secondary tumor apparent regression in H-RT+L-TBI in our study, changes in tumor microenvironment in various groups were evaluated by flow cytometry. Previous studies showed that L-TBI alone can inhibit tumor growth and reduce metastasis in experimental mouse models, mainly by reversing the tumor-associated immune suppression (20, 48). In contrast, L-TBI alone had no effect on tumor growth in our study, and did not significantly reduce MDSCs. However, in H-RT+L-TBI group G-MDSCs and M2 were significantly decreased compare to other groups, as shown in Figure 6. This could be due to the absence of H-RT induced immunogenic tumor cell death. It is reported that if the total dose is split into large doses and administered over a short period of time (8 Gy × 3), they can enhance the immunogenicity (24). As a result, mutual promotion of L-TBI and H-RT activates system anti-tumor immune response.

Demaria et al. showed that abscopal tumor regression was totally dependent on the presence of T cells (49), while Dewan et al. further associated this effect with cytotoxic CD8^+^ T cells (50). Subsequently, several studies showed that T-cells play a crucial role in abscopal tumor regression (51–53). In our research, we also found that H-RT+L-TBI led to the recruitment and activation of T-cells and DCs in the abscopal tumors. This is consistent with the observation that secretory signals of tumor cells might be central for the recruitment of myeloid cells (54, 55). Similarly, DCs also migrate *in vitro* toward irradiated tumor cells, as seen by the increased expression of the activation marker CD86. In our study, the combination treatment resulted in CD8^+^ T-cells and DCs infiltration in the non-irradiated tumor as well, resulting in a markedly attenuation of tumor growth. Furthermore, the anti-tumor CD8^+^ T cells can kill MDSCs via production of TNF-α, IFN-γ, or the expression of apoptotic FasL, and thereby reduce MDSC tumor infiltration (30–32). In our study, its combination with L-TBI increased the number of total CD8^+^ and IFN-γ^+^ CD8^+^ T cells at both secondary tumor and spleen (Figures 5D, 7D). The decrease of CD8^+^cells ended the suppressive effect of the combination therapy at both primary and secondary sites (Supplementary Figures 4B,C). Therefore, the remission of both tumors depended on IFN-γ^+^ CD8^+^ T cells. These findings suggested that CD8^+^ T cells induced by combination therapy were capable of suppressing metastatic and recurrent tumor growth by increasing activated DCs, the level of IFN-γ and the loss of tumor MDSCs. Eosinophil count is increased in a variety of tumors and blood malignancies. The infiltration of eosinophils in the tumor tissue has been associated with improved 5-year survival rate in cancer patients (56). Consistent with this, Eosinophils were significantly increased in the L-TBI+H-RT group, indicating the anti-tumor role of innate immune cells.

Taken together, the combination of H-RT and L-TBI significantly delayed both primary and secondary tumor growth. This approach is more convenient, simpler, and cost-effective compared to RT and IT. Therefore, it is worth studying its underlying mechanisms in greater detail and further testing it in clinical settings. Future optimization of dosing and administration schedule is expected to further increase its efficacy. Our findings highlight the importance of the adaptive immune response in tumor RT and might help to promote the application of low dose RT as a novel approach in treating metastases. In summary, the success of the combination radiation therapy over several weeks in the induction of abscopal remission suggests that CD8^+^T cell infiltration might be the critical factor in controlling the secondary tumor via altering the tumor microenvironment. In addition, the pre-clinical data presented here on the chronology of immune cell infiltration into tumors should help optimize clinical radio-IT protocols.

## Author Contributions

JL, JW, JZ, SL, and SF designed the study and wrote the manuscript. JL, JZ, and MW performed and analyzed the experiments. JL assisted in the establishment of the mouse models and data analysis. CH, JY, DL, PW, YuC, and YoC performed the experiments. YoC, SF, JW, and PC provided critical suggestions and discussions throughout the entire study. JW provided the initial idea of the study.

### Conflict of Interest Statement

The authors declare that the research was conducted in the absence of any commercial or financial relationships that could be construed as a potential conflict of interest.

## Abbreviations

H-RT: hypo-fractionated radiation therapy
L-TBI: low-dose total body irradiation
LET: linear energy transfer
NK: natural killer
MDSCs: myeloid-derived suppressor cells
TAMs: tumor-associated macrophages
Treg: regulatory T-cell
TME: tumor microenvironment
G-MDSCs: granulocytic-myeloid-derived suppressor cells
M- MDSCs: monocytic-myeloid-derived suppressor cells
ROI: irregular region of interest
SUV: standard uptake value.
