# Corrigendum: Low-Dose Total Body Irradiation Can Enhance Systemic Immune Related Response Induced by Hypo-Fractionated Radiation

**DOI:** 10.3389/fimmu.2021.745787

**Published:** 2021-09-03

**Authors:** Jing Liu, Jie Zhou, Min Wu, ChuanFei Hu, Juan Yang, Dong Li, Peng Wu, Yue Chen, Ping Chen, Sheng Lin, YongXia Cui, ShaoZhi Fu, JingBo Wu

**Affiliations:** ^1^Department of Oncology, The Affiliated Hospital of Southwest Medical University, Nuclear Medicine and Molecular Imaging Key Laboratory of Sichuan Province, Luzhou, China; ^2^Department of Radiation Oncology, Sichuan Cancer Hospital and Institute, Sichuan Cancer Center, School of Medicine, University of Electronic Science and Technology of China, Chengdu, China; ^3^Nuclear Medicine and Molecular Imaging Key Laboratory of Sichuan Province, Department of Nuclear Medicine, The Affiliated Hospital of Southwest Medical University, Luzhou, China

**Keywords:** systemic immune related response, hypo-fractionated radiation therapy, low-dose total body irradiation, immune enhancement, immunosuppressive microenvironment

In the original article, there was a mistake in **Figures 3C** and **4C** as published. The authors regret that the wrong images were used. The reason may be owing to layer fusion leading the images to be duplicated (primary tumor and secondary tumor images were repeated in the L-TBI group; secondary tumor images were repeated in the control group and L-TBI group). The corrected **Figures 3** and **4** appear below. Furthermore, the authors have ensured that the arrows now point to identifiable structures within the images. The authors apologize for this error and state that this does not change the scientific conclusions of the article in any way. The original article has been updated.

**Figure 3:**
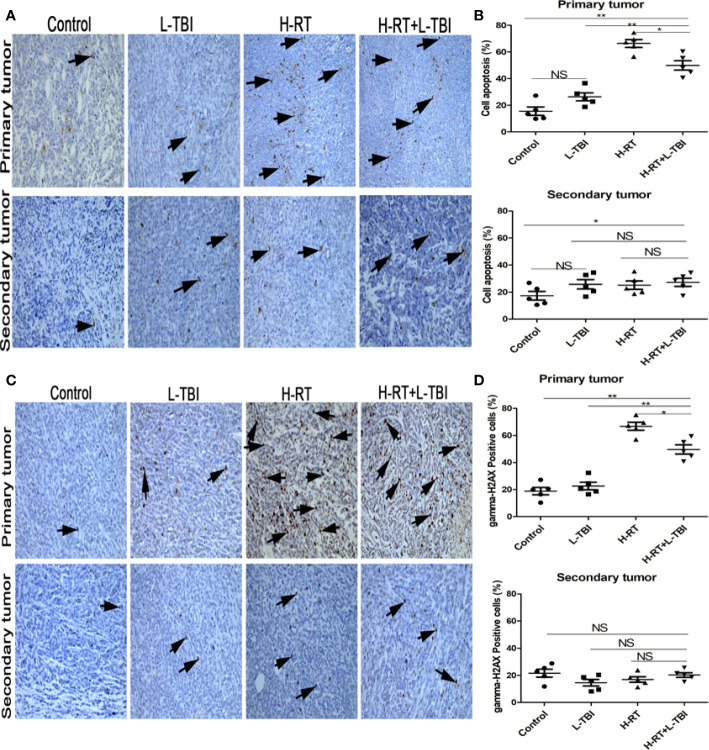
Effect of combination H-RT and L-TBI therapy on apoptosis in 4T1 tumor bearing tissues. **(A)** Comparison of representative TUNEL IHC-stained in different treatment groups. **(B)** Percentage of TUNEL positive cells in the primary and secondary tumor. **(C)** Representative gamma-H2AX IHC staining image in different treatment groups. **(D)** Percentage of gamma H2AX positive cells in the primary and secondary tumor. The arrows point to the TUNEL and gamma-H2AX positive cells in the tumor tissue (original magnification ×200). Data are expressed as mean ± SE of 5 mice/group. (*P < 0.05, **P < 0.01, and N, not significant).

**Figure 4:**
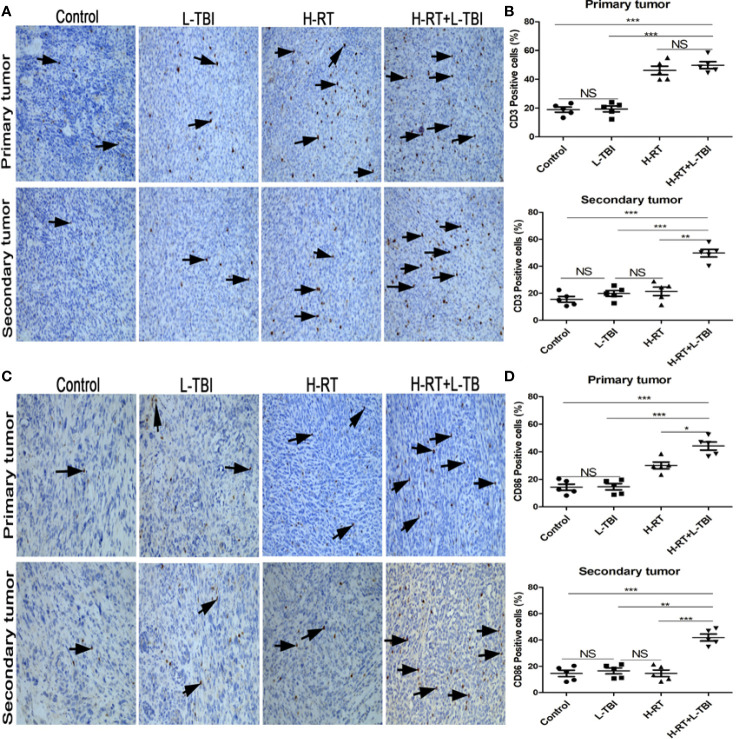
Comparison of CD3+ and CD86+ lymphocytes in different treatment groups. **(A)** Representative images of CD3 IHC in tumor tissues of different treatment groups. **(B)** Percentage of CD3 positive cells in the primary and secondary tumor. **(C)** Representative IHC images of CD86 infiltration in the tumor tissue of different treatment groups. **(D)** Percentage of CD86 positive cells in the primary and secondary tumor. The arrows point the CD3 and Cd86 positive cells in tumor tissues from mice that received different treatments (original magnification ×200). Data are expressed as mean ± SE of 5 mice/group. (*P < 0.05, **P < 0.01, ***P < 0.001, and NS, not significant).

## Publisher’s Note

All claims expressed in this article are solely those of the authors and do not necessarily represent those of their affiliated organizations, or those of the publisher, the editors and the reviewers. Any product that may be evaluated in this article, or claim that may be made by its manufacturer, is not guaranteed or endorsed by the publisher.

