# Supplementary material for: Low-Dose Total Body Irradiation Can Enhance Systemic Immune Related Response Induced by Hypo-Fractionated Radiation
Source: Front Immunol. 2019 Feb 27;10:317. doi: 10.3389/fimmu.2019.00317 (PMC6401363; doi:10.3389/fimmu.2019.00317)
Supplement: Supplementary file 1 [file Data_Sheet_1.PDF]

Supplementary Material

Low-dose total body irradiation can enhance systemic immune related response induced by hypo-fractionated radiation

Jing Liu[a†], Jie Zhou[b†], Min Wu[a†], ChuanFei Hu[a], Juan Yang[a], Dong Li[a], Peng Wu[a], Yue Chen[c], Ping Chen[a], Sheng Lin[a], YongXia Cui[a], ShaoZhi Fu[a,*], JingBo Wu[a,*]

***Correspondence:** JingBo Wu; E-mail: wjb6147@163.com; ShaoZhi Fu; E-mail: shaozhifu513@163.com.

Supplementary Figures

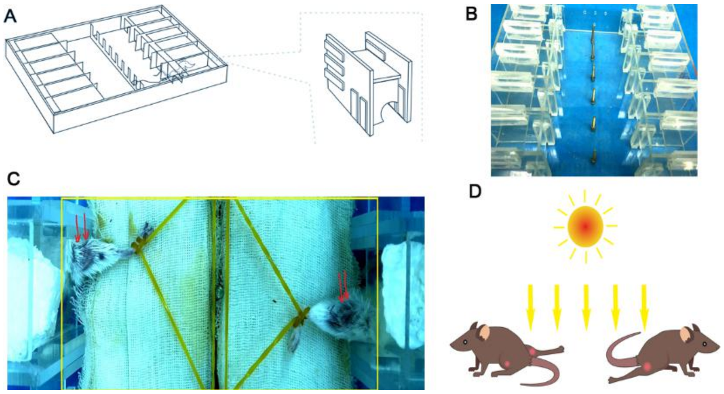

Supplementary Figure 1. Irradiation protocol. The mouse was fixed using a RT box **(A and B)**. The right leg and primary tumor were within the radiation field. **(C)** The fixed form (yellow solid line area representing the radiation field). **(D)** The aim of the exposure.

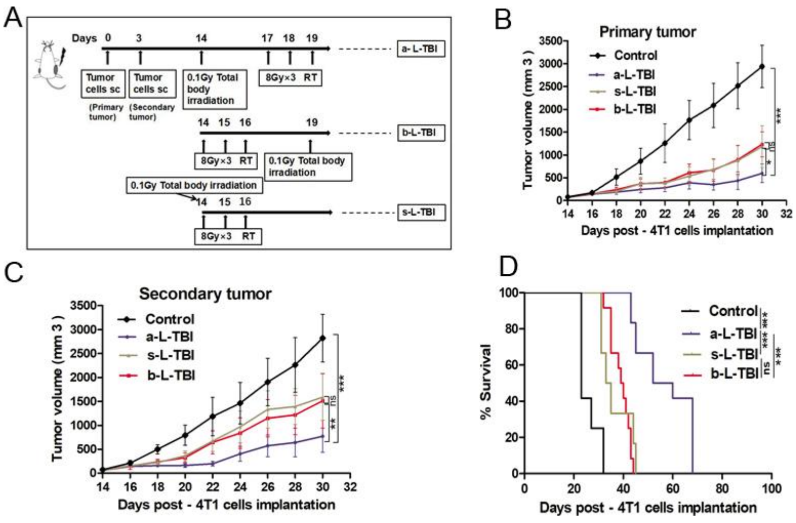

Supplementary Figure 2. Effect of different sequence of the H-RT and L-TBI combination therapy in 4T1 tumor-bearing mice. **(A)** Treatment timeline of the 4T1 mammary carcinoma BALB/C mouse model. a-L-TBI represents that we administered local H-RT after L-TBI. s-L-TBI represents that we simultaneously administered local H-RT and L-TBI. b-L-TBI represents that we administered local H-RT before L-TBI. Tumor growth of primary tumors **(B)** and secondary tumor **(C)** in different experimental groups. **(D)** Overall survival curves of the treatment groups. Data are expressed as mean ± SE of 12 mice/group. The statistical significance of differences was determined by ANOVA. (**P* <0.05, ***P* <0.01, ****P* <0.001, NS = not significant).

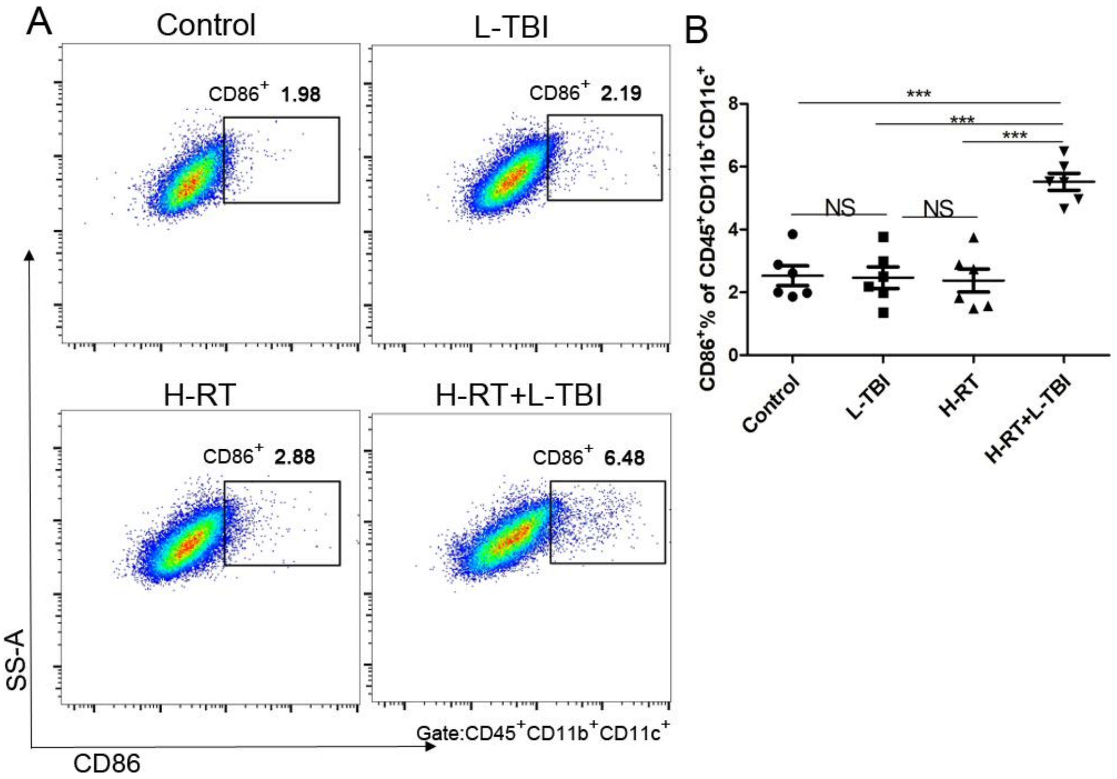

Supplementary Figure 3. Expression of $CD86^+$ cells on DCs in mice treated with H-RT and L-TBI radiotherapy. **(A)** Representative dot plots of $CD86^+$DCs ($CD45^+CD11b^+CD11c^+CD86^+$) cells in the secondary tumor tissue of control, L-TBI, H-RT, and H-RT+L-TBI group. **(B)** Comparison plot of $CD86^+$DCs cells in the secondary tumor tissue of different groups. Data are representative charts or the percentages of individual subjects. The lines indicate median values for each group. Data are expressed as mean ± SE of 6 mice/group (**P* <0.05, ***P* <0.01, ****P* <0.001, NS = not significant).

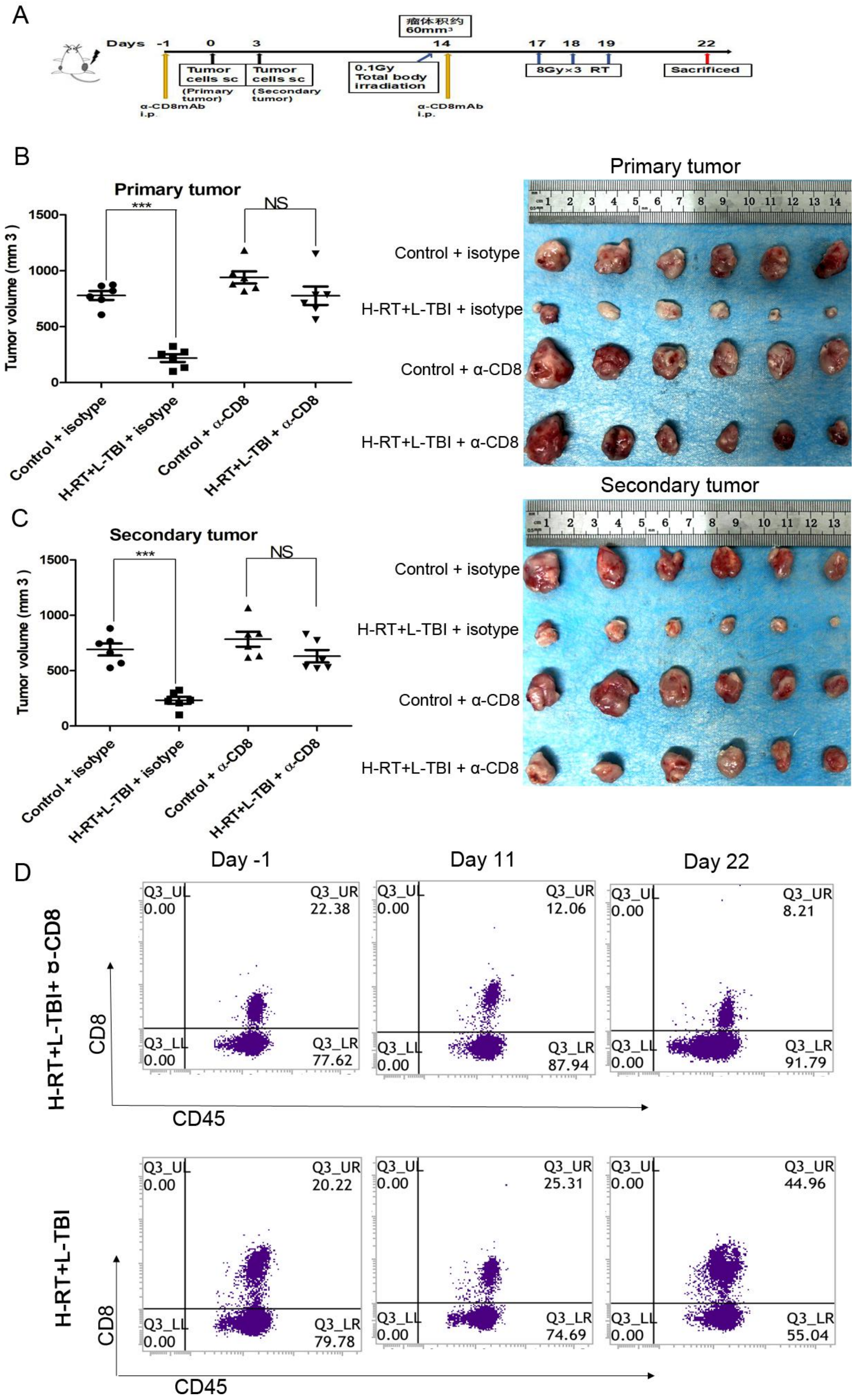
A
Days -1 0 3 14 17 18 19 22
瘤体积约 60mm³
Tumor cells sc (Primary tumor)
Tumor cells sc (Secondary tumor)
0.1Gy Total body irradiation
8Gy×3 RT
Sacrificed
α-CD8mAb i.p.
α-CD8mAb i.p.
B
Primary tumor
NS
Tumor volume (mm 3)
Control + isotype
H-RT+L-TBI + isotype
Control + α-CD8
H-RT+L-TBI + α-CD8
C
Secondary tumor
NS
Tumor volume (mm 3)
Control + isotype
H-RT+L-TBI + isotype
Control + α-CD8
H-RT+L-TBI + α-CD8
D
Day -1
Day 11
Day 22
H-RT+L-TBI+ α-CD8
CD8
CD45
Q3_UL 0.00
Q3_UR 22.38
Q3_LL 0.00
Q3_LR 77.62
Q3_UR 12.06
Q3_LR 87.94
Q3_UR 8.21
Q3_LR 91.79
H-RT+L-TBI
Q3_UR 20.22
Q3_LR 79.78
Q3_UR 25.31
Q3_LR 74.69
Q3_UR 44.96
Q3_LR 55.04

1

Supplementary Figure 4. The decrease of $CD8^+$ cells abrogated the antitumor effect of the H-RT and L-TBI combination therapy on primary and secondary tumor. **(A)** Treatment timeline of the 4T1 mammary carcinoma BALB/C mouse model. Anti-CD8/Lyt2.1 monoclonal antibody or mouse IgG2a was injected on day –1 and 14. 4T1-bearing mice (n = 6 mice/group) were treated with the combination therapy. Tumor volume and pictures on day 22 of Control (+isotype/α-CD8) and the H-RT and L-TBI combination therapy (+isotype/α-CD8) on primary tumor **(B)** and secondary tumor **(C)**. **(D)** Representative dot plots of $CD8^+$ in blood from mice treated with H-RT+L-TBI+α-CD8 and H-RT+L-TBI on days -1, 11, and 22. Data are representative charts or the percentages of individual subjects. The lines indicate median values for each group (*P<0.05, **P<0.01, ***P<0.001, NS = not significant).

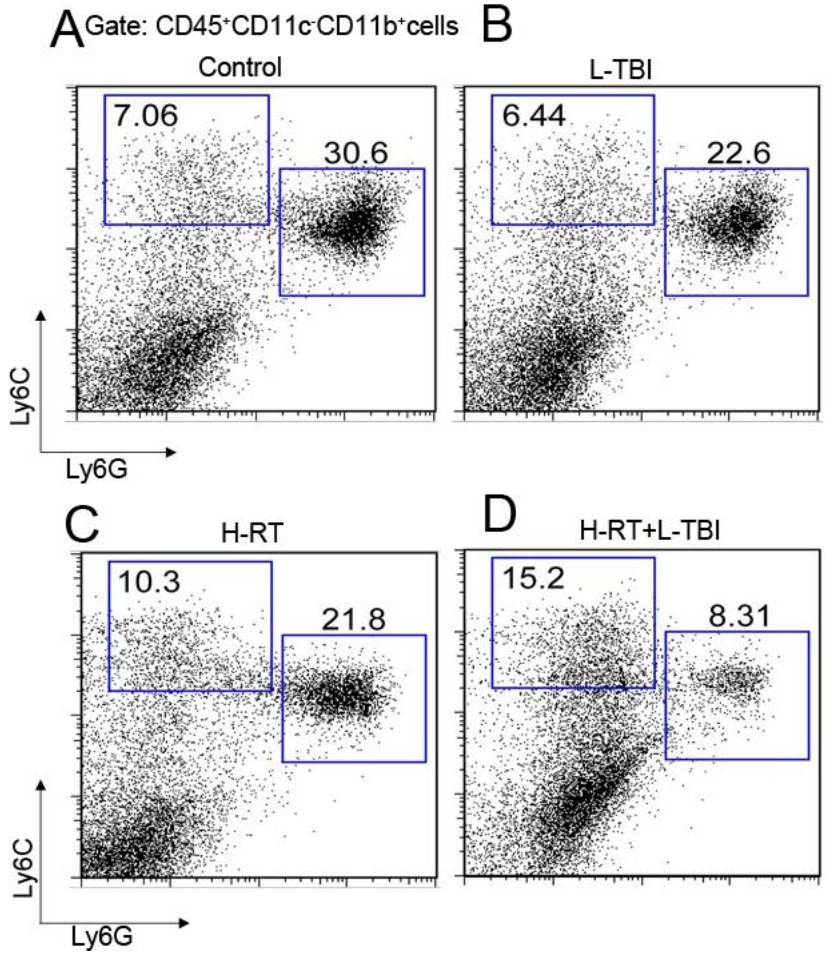

Supplementary Figure 5. H-RT and L-TBI combination resulted in the reduction of G-MDSCs in the secondary 4T1 tumor. Representative dot plots showing G-MDSCs and M-MDSCs in the secondary tumor tissue of **(A)** Control, **(B)** L-TBI, **(C)** H-RT, and **(D)** H-RT+L-TBI group (n=5 mice/group).

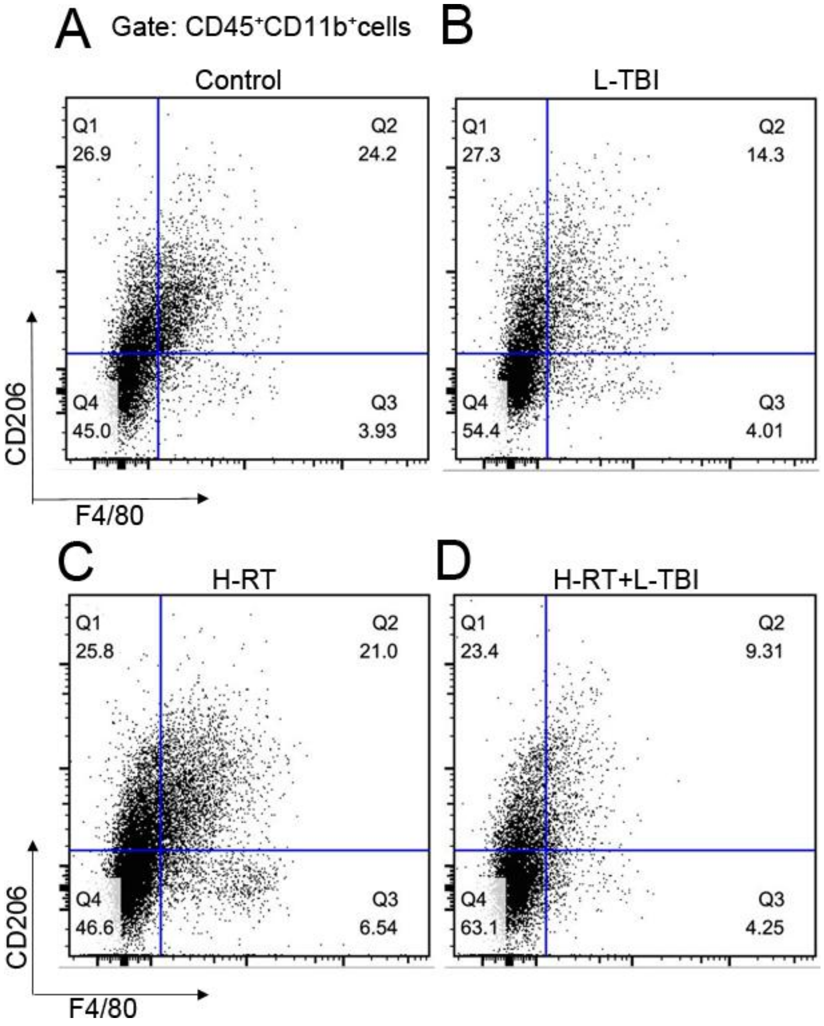

Supplementary Figure 6. H-RT and L-TBI combination resulted in the reduction of M2 in the secondary 4T1 tumor. Representative dot plots showing M1and M2 in the secondary tumor tissue of **(A)** Control, **(B)** L-TBI, **(C)** H-RT, and **(D)** H-RT+L-TBI group (n=5 mice/group).

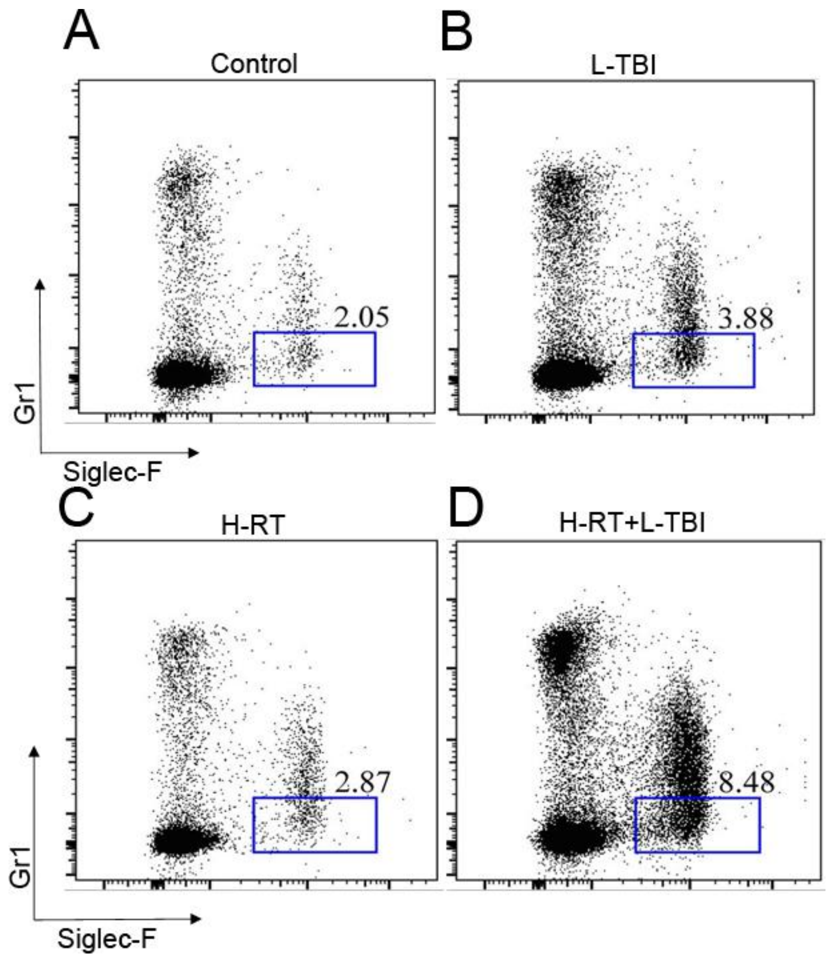

Supplementary Figure 7. H-RT and L-TBI combination resulted in the increase of eosinophils in the secondary 4T1 tumor. Representative dot plots showing Eosinophils in the secondary tumor tissue of **(A)** Control, **(B)** L-TBI, **(C)** H-RT, and **(D)** H-RT+L-TBI group (n=5 mice/group).

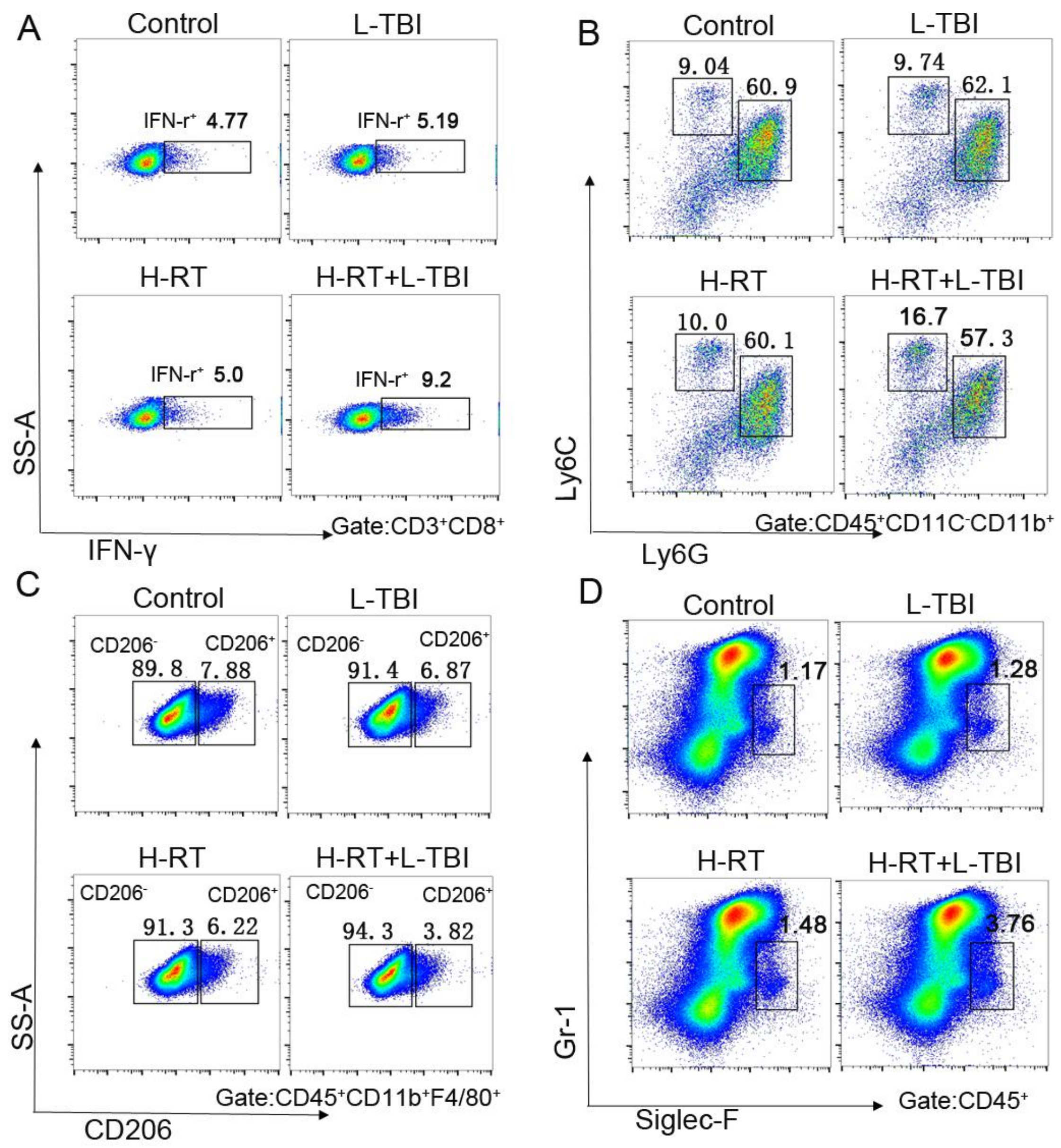

Supplementary Figure 8. Spleens from mice were analyzed for the expression of IFN-γ^+ CD8$^+$T cells, MDSC, TAM, and eosinophils by flow cytometry (n=6 mice/group). Representative dot plots of IFN-γ^+CD8$^+$ T cells **(A)**, MDSC **(B)**, TAM **(C)**, and Eosinophils **(D)** in the spleen of Control, L-TBI, H-RT, and H-RT+L-TBI group. Data are representative charts or the percentages of individual subjects.
